# Voltage distribution over capacitively coupled plasma electrode for atmospheric-pressure plasma generation

**DOI:** 10.1186/1556-276X-8-202

**Published:** 2013-05-01

**Authors:** Mitsutoshi Shuto, Fukumi Tomino, Hiromasa Ohmi, Hiroaki Kakiuchi, Kiyoshi Yasutake

**Affiliations:** 1 Graduate School of Engineering, Osaka University, 2-1 Yamada-oka, Suita, Osaka, 565-0871, Japan; 2 FTR Co. Ltd., 3-18-63 Tsukushino, Machida, Tokyo, 194-0001, Japan

**Keywords:** CCP, Atmospheric-pressure plasma CVD, TLM, Voltage distribution

## Abstract

When capacitively coupled plasma (CCP) is used to generate large-area plasma, the standing wave effect becomes significant, which results in the hindering of the uniform plasma process such as in a plasma etcher or plasma chemical vapor deposition. In this study, the transmission line modeling method is applied to calculate the voltage distribution over atmospheric-pressure CCP electrodes with the size of 1 m × 0.2 m. The measured plasma impedance in our previous study was used in the present calculation. The results of the calculations clearly showed the effects of excitation frequency and the impedance of the plasma on the form of the voltage distribution caused by the standing wave effect. In the case of 150 MHz frequency, the standing wave effect causes a drastic change in the voltage distribution via plasma ignition; however, the change is small for 13.56 MHz. It was also clarified that the power application position is important for obtaining a uniform voltage distribution.

## Background

Plasma-enhanced chemical vapor deposition (PECVD) is an important and widely used process for forming various kinds of thin films in the electronics industry to fabricate, for example, very-large-scale integration and solar cells. For PECVD, capacitively coupled plasma (CCP) has the advantage of generating the large-area plasma necessary to process large substrates. However, when the electrodes become large relative to the wavelength of the electromagnetic wave used to generate the plasma, the standing wave effect will become significant, deteriorating the uniformity of the film thickness obtained [1-5].

It is considered that the voltage distribution over the CCP electrode greatly affects not only the distribution of plasma characteristics, such as plasma density and electron temperature, but also the deposited film thickness uniformity, especially in the case of PECVD. In this study, the voltage distribution over the CCP electrode was calculated by the transmission line modeling (TLM) method [6] for 13.56 and 150 MHz and with different power application positions. A two-dimensional (1 m × 0.2 m) plane electrode was modeled, and the impedances of the atmospheric-pressure plasma obtained from IV (current and voltage) measurements and analysis [7] were used for the calculation.

## Methods

### Modeling

**Figure 1:**
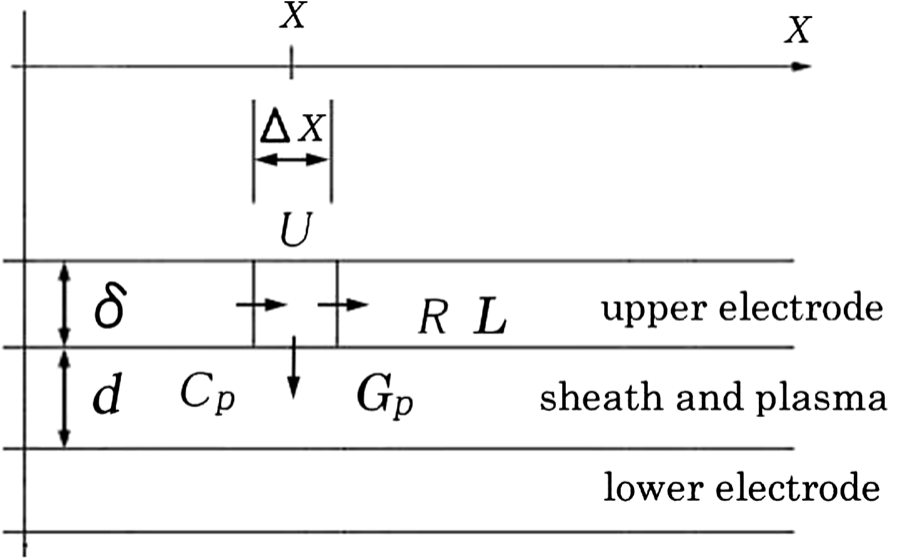
One-dimensional model of electrodes and plasma (including sheath).

A one-dimensional model of electrodes and plasma (including sheath) is shown in Figure 1. Radio-frequency voltage is applied to the upper electrode, and the lower electrode is grounded. We assume that only the upper electrode has resistance and inductance for simplicity. This simplified model is useful enough to calculate a relative voltage between two electrodes, because only relative voltage is important for plasma.

Plasma will be generated in the space between the upper and lower electrodes. In this model, electrodes (upper and lower) and plasma are divided into small elements of length Δ*X*. The voltage *U* is assumed to be constant within the elements. Symbol *δ* is the thickness of current flow (skin depth). The currents flowing into and out from the element are shown by the arrows in Figure 1. The plasma is assumed to be able to be represented by the parallel connection of the capacitance *C*_p_ and the conductance *G*_p_.

One can derive a one-dimensional wave equation from the above mentioned one-dimensional model and extend it to the following two-dimensional wave equation. In the case of a two-dimensional model, the electrode will be divided in two directions, and the widths of the element will be Δ*X* and Δ*Y*. For simplicity, the element widths Δ*X* and Δ*Y* were set to be equal.

(1) $$\frac{\partial^{2}U}{\partial t^{2}}=\frac{1}{LC_{p}}\left(\frac{\partial^{2}U}{\partial x^{2}}+\frac{\partial^{2}U}{\partial y^{2}}\right)-\frac{RC_{p}+LG_{p}}{LC_{p}}\frac{\partial U}{\partial t}-\frac{RG_{p}}{LC_{p}}U+F\left(x,y,t\right)$$

Here, *L* and *R* are inductance and resistance per unit length (in current flow direction) of the electrodes of element width (Δ*X* or Δ*Y*), and *C*_p_ and *G*_p_ are the capacitance and conductance of plasma per unit length of element width, respectively. *F*(*x*,*y*,*t*) is the external force (causes voltage to change) applied to the upper electrode at position (*x*,*y*).

### Electrode resistance *R* and inductance *L*

When radio-frequency power is applied to the electrodes, the current will flow only on the surface of the electrodes owing to the skin effect. The effective electrode resistance per unit length *R* (of width *w*) is determined by the following equation [8]:

(2) $$R=\frac{2}{\mathrm{\sigma \delta w}},$$

where *σ* is the conductivity of the electrode material, *δ* is the skin depth, and *w* is the width of the current flow. The skin depth *δ* is determined by

(3) $$\delta =\sqrt{\frac{2}{\sigma \omega \mu }},$$

where *ω* is the angular frequency, and *μ* is the magnetic permeability of the electrode material.

The inductance of a pair of two parallel plates (electrodes) per unit length (of width *w*) can be calculated using [8]

(4) $$L=\mu \frac{d}{w},$$

where *d* is the distance between the upper and lower electrodes, and *w* is the width of the current flow. When aluminum is used as the electrode material, the conductivity *σ* is 0.33 × 10^8^ ohm^−1^ m^−1^.

Table 1 shows a summary of these values calculated for the cases of 150 MHz and 13.56 MHz.

**Table 1:** **Effective resistances and inductances of the Al electrode element**[6]

	**150 MHz**	**13.56 MHz**
*R* (ohm/m)	0.843	0.253
*L* (H/m)	1.26 × 10^−7^	1.26 × 10^−7^

The element width is 0.01 m.

### Plasma conductance *G*_p_ and capacitance *C*_p_

**Figure 2:**
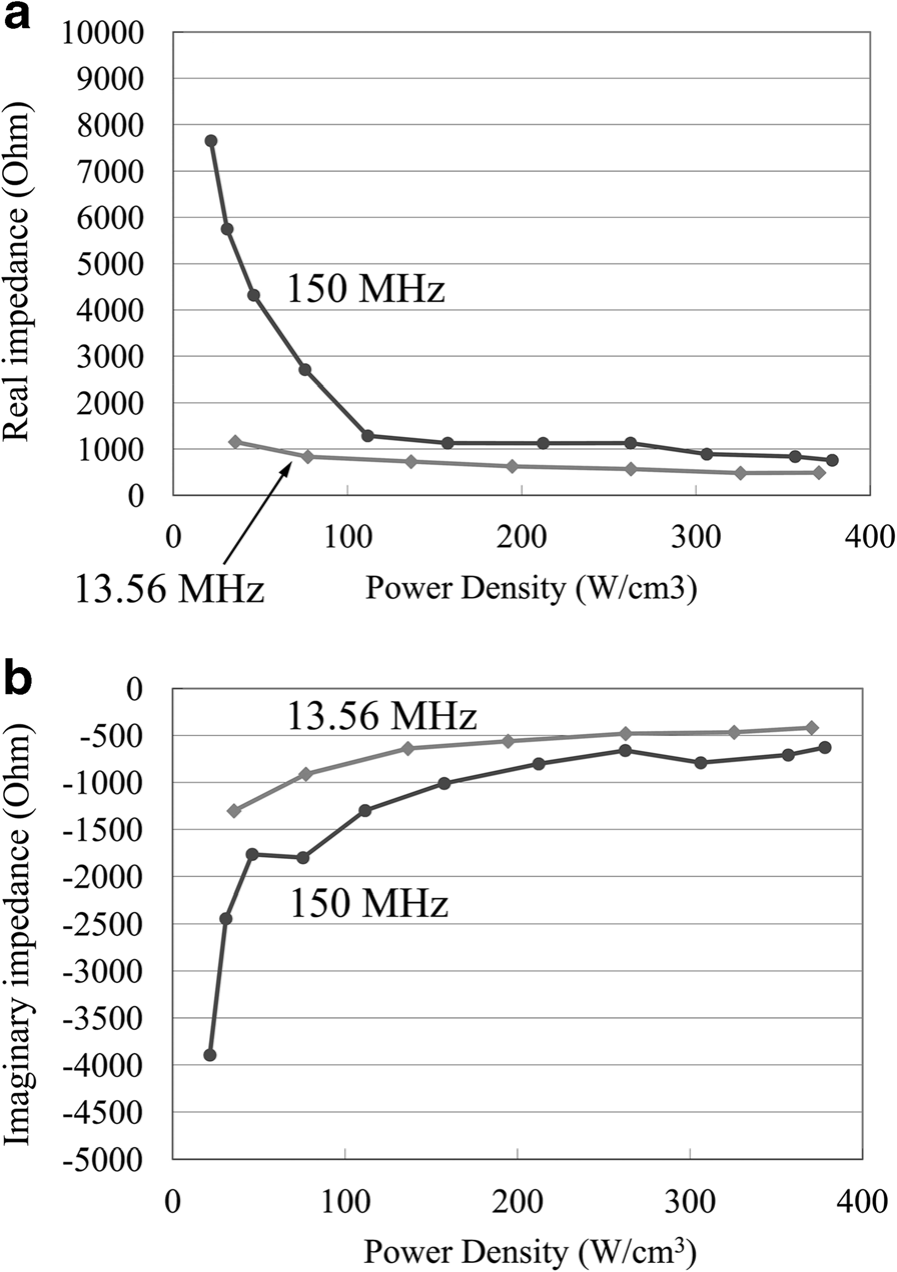
**Real (a) and imaginary (b) parts of plasma impedance vs. applied power density.** Electrode diameter, 1 cm; electrode gap, 1 mm.

In the case of atmospheric-pressure plasma, since the gap between the electrodes is usually too narrow (≤1 mm) to perform Langmuir probe analysis, we performed plasma impedance analysis in our previous study [7]. A combination of the measurement of the current and voltage waveforms outside of the apparatus and calculation using the electrically equivalent circuit model enabled us to derive the impedance *Z*_p_ of the plasma-filled capacitor. Figure 2 shows the measured impedance of atmospheric-pressure helium plasma (real (Figure 2a) and imaginary (Figure 2b) parts of *Z*_p_) as a function of applied power density, for 150 MHz and 13.56 MHz excitations using a metal electrode with a diameter of 10 mm and a gap of 1 mm. As shown in Figure 2, the plasma impedance *Z*_p_ changes depending on the applied power; this is known as a nonlinear characteristic of the plasma. However, it is also shown that the impedance becomes constant (the system is linear) in a considerably wide power range when sufficiently high power is applied to the plasma. Although taking the nonlinear characteristic of plasma into account will give more exact results, we consider that it is still meaningful to calculate the voltage distribution on the assumption that the plasma impedance is constant, since plasma equipment is often used in such a saturated area.

The plasma conductance *G*_p_ and the susceptance *B*_p_ per unit length of element width are calculated from a given plasma impedance *Z*_p_ (*Z*_p_ = *R*_p_' − *X*_p_*j*) using

(5) Gp=Rp'Rp'2+Xp2,

(6) $$B_{p}=\frac{X_{p}}{R_{p}{'}^{2}+X_{p}{}^{2}}.$$

**Figure 3:**
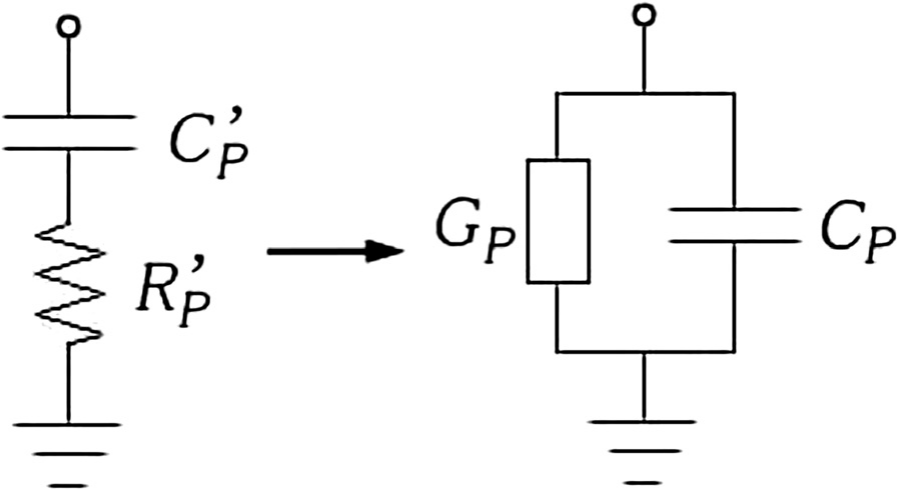
Conversion of plasma impedance (left) to admittance (right).

Then the plasma (parallel) capacitance *C*_p_ per unit length of element width at a particular frequency *ω* (shown in Figure 3) can be calculated from plasma susceptance *B*_p_, as

(7) $$C_{p}=\frac{B_{p}}{\omega }=\frac{X_{p}}{\omega \left(R_{p}{'}^{2}+X_{p}{}^{2}\right)}.$$

### Wavelength and phase velocity in the electrodes

The propagation constant *γ* ≡ *α* + *βj* of the solution of Equation 1 is

(8) $$\gamma =\sqrt{\left(R+\omega \;\mathrm{Lj}\right)\left(G_{p}+\omega \;C_{p}j\right)}.$$

Its real part *α* (attenuation coefficient) and imaginary part *β* (phase propagation constant) are described as

(9) $$\alpha =\sqrt{\frac{1}{2}\left\{\sqrt{\left(R^{2}+\omega^{2}L^{2}\right)\left(G_{p}{}^{2}+\omega^{2}C_{p}{}^{2}\right)}+\left(RG_{p}-\omega^{2}LC_{p}\right)\right\}}$$

and

(10) $$\beta =\sqrt{\frac{1}{2}\left\{\sqrt{\left(R^{2}+\omega^{2}L^{2}\right)\left(G_{p}{}^{2}+\omega^{2}C_{p}{}^{2}\right)}-\left(RG_{p}-\omega^{2}LC_{p}\right)\right\}}.$$

The phase velocity *v* of the electromagnetic wave propagating in the system described by Equation 1 is

(11) $$v=\frac{\omega }{\beta }.$$

The wavelength *λ* is calculated using

(12) $$\lambda =\frac{2\pi }{\beta }.$$

From these equations, it is clear that the wavelength on the electrode is governed not only by the electrode configuration but also the impedance of plasma. Both the attenuation coefficient *α* and the wavelength *λ* greatly affect how a standing wave is formed on the electrode.

## Results and discussion

Equation 1 can be numerically solved by a finite differential method. Calculation was performed for an electrode size of 0.2 × 1 m^2^, with the edges of the electrodes assumed to be open. Usually, plasma equipment is designed so that the edge of the electrode is not exposed to the plasma. Sometimes, the edges of the electrode will be supported by dielectric materials such as quartz and ceramics, in which case the edges are terminated by the capacitance formed by the dielectrics. In such a case, in order to minimize the power loss, the electrode supporting system will be designed so that the capacitance becomes as small as possible, in which case the impedance is close to that of the open case. The electrode was divided into small elements of which the size is 0.01 × 0.01 m (Δ*X* = Δ*Y* = 0.01 m). Both *C*_p_ and *G*_p_ are assumed to stay constant with relatively small variation in the electrode voltage. *C*_p_ and *G*_p_ values were calculated from the measured impedance of atmospheric-pressure helium plasma (*Z*_p_) shown in Figure 2.

Table 2 shows the plasma impedance *Z*_p_, admittance *Y*_p_, and (parallel) capacitance *C*_p_ used for the calculations. The propagation constant γ and the wavelength λ are also shown. It is seen that the wavelength λ on the electrode is considerably shorter than that in free space.

**Table 2:** **Measured impedances of atmospheric-pressure helium plasma**[7]

	**150 MHz (378.2 W/cm**^**3**^**)**	**13.56 MHz (370.5 W/cm**^**3**^**)**
*Z*_p_ = *R*_p_*′ + X*_p_*j* (ohm/m^2^)	0.060 – 0.049 *j*	0.038 – 0.033 *j*
*Y*_p_ = *G*_p_*+ B*_p_*j* (1/(ohm m^2^))	9.96 + 8.25 *j*	15.0 + 13.0 *j*
*C*_p_ (F/m^2^)	8.75 × 10^−9^	1.53 × 10^−7^
*γ* ≡ *α* + *βj*	1.69 + 3.54 *j*	0.62 + 1.32 *j*
*λ*(m)	1.77 (2 m in free space)	4.78 (22.1 m in free space)

Electrode diameter, 1 cm; electrode gap, 1 mm.

**Figure 4:**
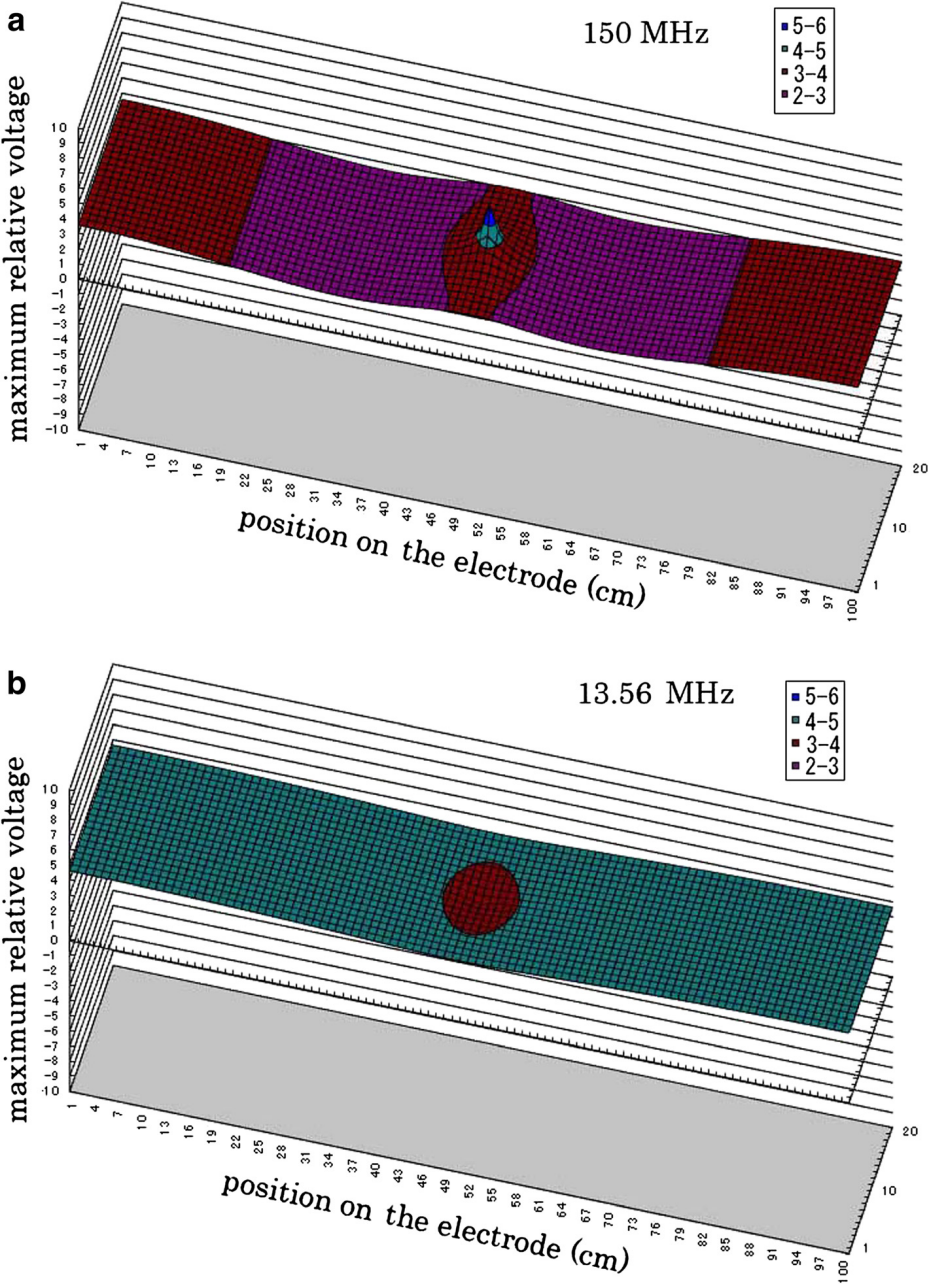
**Two-dimensional distribution of voltage amplitude on the electrode during plasma generation.** Power was applied at the center of the electrode. (**a**) 150 MHz and (**b**) 13.56 MHz.

Figure 4 shows the calculated two-dimensional distribution of the voltage amplitude at each point on the electrode during plasma generation. The power was applied at the center of the electrode.

**Figure 5:**
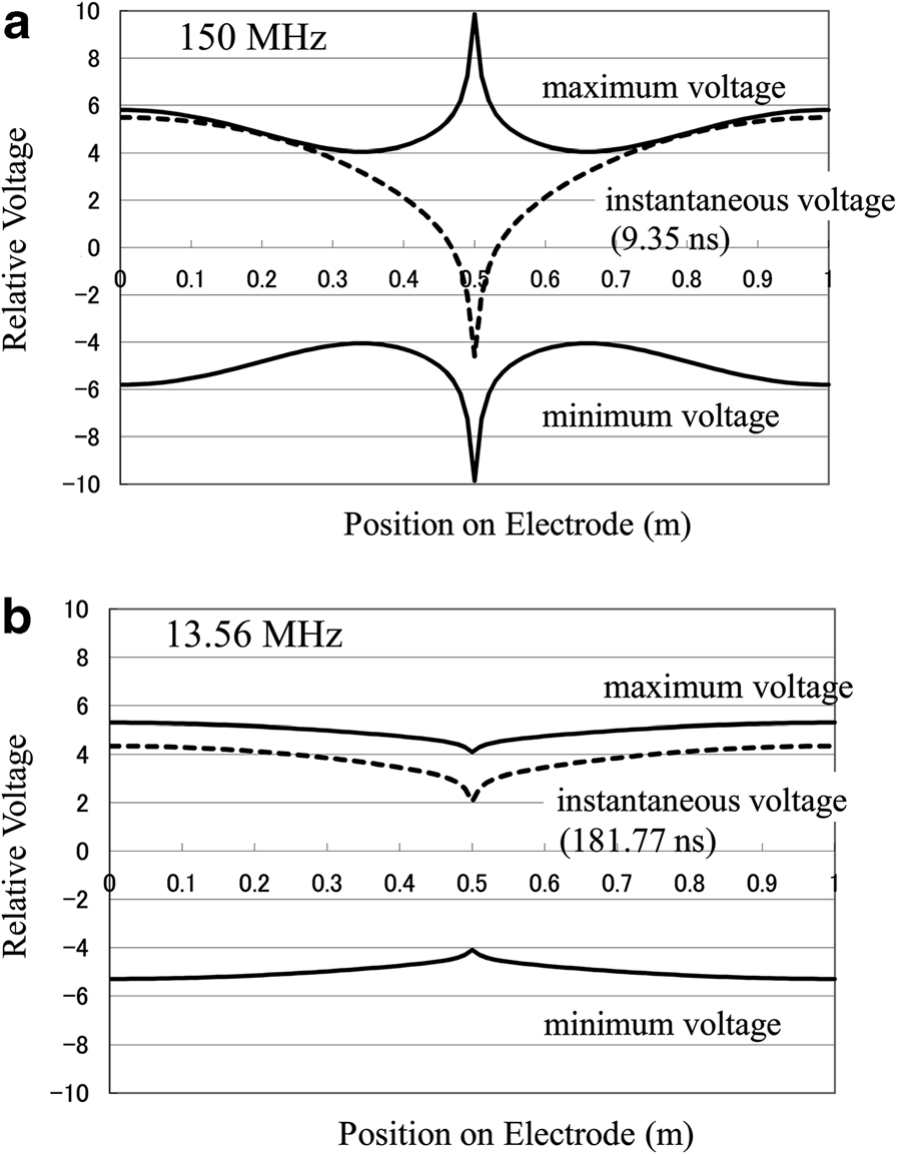
**Voltage distributions along the central cross-sectional line on the electrode.** Power was applied at the center of the electrode. (**a**) 150 MHz and (**b**) 13.56 MHz.

The central cross-sectional distributions of the plots in Figure 4 are shown in Figure 5, where voltage distribution is along the central cross-sectional line in the direction of electrode length. Voltages oscillate between their maximum and minimum with the driving frequency. Dotted lines in Figure 5 show instantaneous voltage profiles at elapsed times of 9.35 and 181.77 ns for 150 and 13.56 MHz, respectively. They always remain between the maximum voltage (upper solid line) and the minimum voltage (lower solid line). It is clearly seen that voltage variation is considerably larger for 150 MHz than for 13.56 MHz. The voltage variation over the electrode is approximately 58% and 12% for 150 and 13.56 MHz, respectively.

The spatial differentiation of the instantaneous voltage gives the electric field in the horizontal direction at the differentiation point. As shown in Figure 5, the gradient of the instantaneous voltage is largest at the driving point. According to the calculation, the largest gradient of the instantaneous voltage in 150 MHz case was approximately 0.45 V/m, while the average electric field across the electrodes was 5,000 V/m. This means that the current flowing in the horizontal direction is small enough compared with that flowing in the vertical direction. Since the difference was even larger in the 13.56 MHz case, the current flowing in the horizontal direction can be neglected.

**Figure 6:**
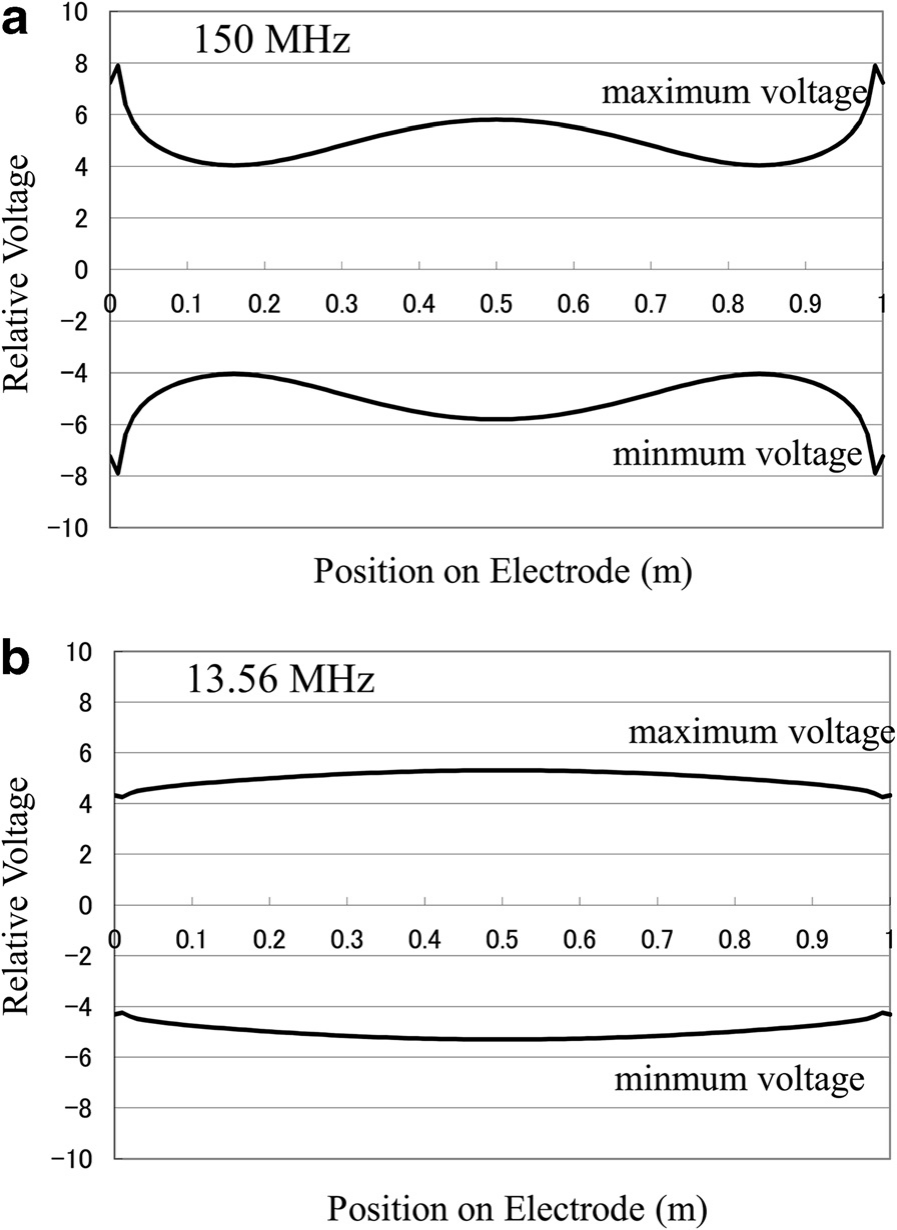
**Voltage distributions along the central cross-sectional line on the electrode during plasma generation.** Power was applied on both ends of the electrode with the same phase. (**a**) 150 MHz and (**b**) 13.56 MHz.

Very different voltage distribution profiles are obtained when radio-frequency power is applied on both ends of the electrode, as shown in Figure 6. The phase of radio frequency was set to be the same. The voltage variations over the electrode are approximately 39% and 11% for 150 and 13.56 MHz, respectively. Therefore, this type of power application would be more advantageous for obtaining more uniform plasma over the electrode.

**Figure 7:**
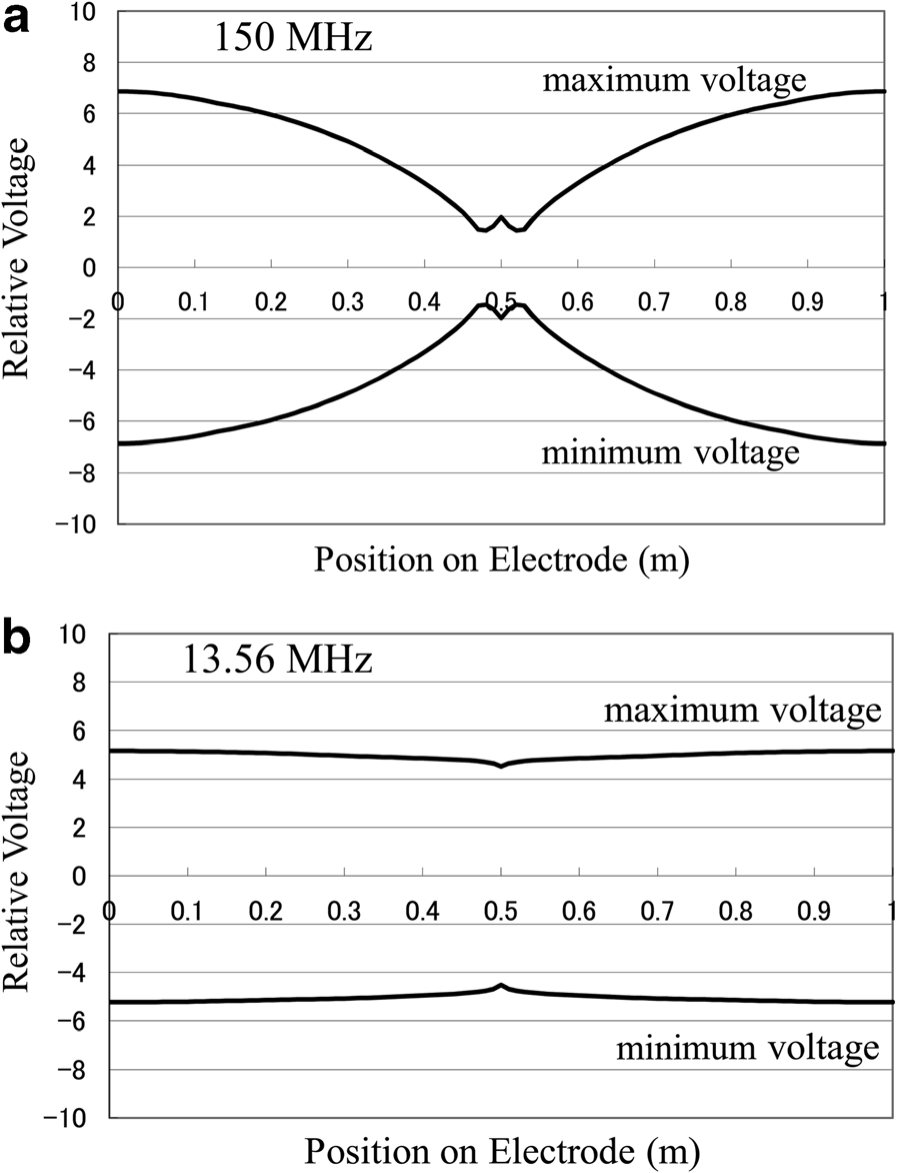
**Voltage distribution on the electrode before plasma ignition.** Power was applied at the center of the electrode. (**a**) 150 MHz and (**b**) 13.56 MHz.

Figure 7 shows the results of the calculations of voltage distribution before plasma ignition. When there is no plasma between the electrodes, the conductance *G* is zero and the capacitance *C* is determined by

(13) $$C=\frac{\epsilon_{0}S}{d},$$

where ϵ_0_ is the permittivity of vacuum. *S* and *d* are the electrode area and the distance between the upper and lower electrodes, respectively.

Comparing Figure 7 with Figure 5, a slight difference is seen in the case of 13.56 MHz. When 150 MHz is applied, however, the voltage distribution before plasma ignition is considerably different from that after plasma ignition.

From the attenuation coefficient *α* shown in Table 2, the resistive loss in the 150 MHz case is larger than that in the 13.56 MHz case. However, the resistive loss only causes a monotonic decay in voltage amplitude from the driving point along the wave-propagation direction. Since Figure 5 does not show a monotonic decay in voltage from the driving point, the drastic change in the voltage pattern in the 150 MHz case is considered to be caused mainly by the standing wave effect.

The interference pattern may change sensitively with the changes in various parameters (e.g. electrode shape, setup, and plasma parameters) in the case of 150 MHz. It can be said that in the case of 13.56 MHz, the expected or measured voltage distribution before plasma ignition is useful for designing the electrode setup. However, in the case of 150 MHz, careful design of the electrode setup should be required to obtain stable and uniform plasma generation.

## Conclusions

A mathematical model for calculating the voltage distribution over a parallel-plate plasma electrode was constructed by the TLM method. In this study, driving frequencies of 150 MHz and 13.56 MHz were compared. Actually measured atmospheric-pressure helium plasma impedance was used for these calculations. In the case of 150 MHz frequency, the standing wave effect caused a drastic change in the voltage distribution on the electrode by plasma ignition; however, the change was small for 13.56 MHz. Thus, in the case of 13.56 MHz, the expected or measured voltage distribution before plasma ignition is useful for designing the electrode setup. However, in the case of 150 MHz, careful design of the electrode setup should be required to obtain stable and uniform plasma generation. It was also shown that the power application position is important for obtaining uniform voltage distribution. It is considered that the voltage distribution will greatly affect the plasma density distribution and therefore film thickness uniformity in the case of plasma CVD. The TLM method is applicable to circular electrodes as well, and not only to atmospheric-pressure plasma but also to low-pressure plasma. The simulation by the TLM method will be useful in optimizing the configurations of parallel-plate plasma systems.

## Competing interests

The authors declare that they have no competing interests.

## Authors’ contributions

MS developed the finite differential element code, ran the simulations, and wrote the manuscript. FT, HO, HK, and KY assisted in designing the work, discussed the results, and proofread the manuscript. All the authors read and approved of the final manuscript.
